# Artificial intelligence and digital biomarker in precision pathology guiding immune therapy selection and precision oncology

**DOI:** 10.1002/cnr2.1796

**Published:** 2023-02-22

**Authors:** Ralf Huss, Johannes Raffler, Bruno Märkl

**Affiliations:** ^1^ Medical Faculty University Augsburg Augsburg Germany; ^2^ Institute for Digital Medicine University Hospital Augsburg Augsburg Germany

**Keywords:** artificial intelligence, decision support, digital biomarker, immune oncology, precision pathology

## Abstract

**Background:**

The currently available immunotherapies already changed the strategy how many cancers are treated from first to last line. Understanding even the most complex heterogeneity in tumor tissue and mapping the spatial cartography of the tumor immunity allows the best and optimized selection of immune modulating agents to (re‐)activate the patient's immune system and direct it against the individual cancer in the most effective way.

**Recent Findings:**

Primary cancer and metastases maintain a high degree of plasticity to escape any immune surveillance and continue to evolve depending on many intrinsic and extrinsic factors In the field of immune‐oncology (IO) immune modulating agents are recognized as practice changing therapeutic modalities. Recent studies have shown that an optimal and lasting efficacy of IO therapeutics depends on the understanding of the spatial communication network and functional context of immune and cancer cells within the tumor microenvironment. Artificial intelligence (AI) provides an insight into the immune‐cancer‐network through the visualization of very complex tumor and immune interactions in cancer tissue specimens and allows the computer‐assisted development and clinical validation of such digital biomarker.

**Conclusions:**

The successful implementation of AI‐supported digital biomarker solutions guides the clinical selection of effective immune therapeutics based on the retrieval and visualization of spatial and contextual information from cancer tissue images and standardized data. As such, computational pathology (CP) turns into “precision pathology” delivering individual therapy response prediction. Precision Pathology does not only include digital and computational solutions but also high levels of standardized processes in the routine histopathology workflow and the use of mathematical tools to support clinical and diagnostic decisions as the basic principle of a “precision oncology”.

## 1 INTRODUCTION

There is already a significant number of publications using AI and deep learning (DL) to identify novel diagnostic and prognostic biomarker signatures on tissue images of different cancer types.^1^, ^2^, ^3^, ^4^, ^5^, ^6^ Understanding the morphological and immunological complexity and plasticity of the cancer‐related immune system in tissue is still one of the existing challenges in cancer immunotherapy.^7^, ^8^, ^9^, ^10^, ^11^ The visualization of any contextual and spatial relationship of different immune, tumor and stromal cells, the communication network including humoral (extrinsic) and molecular (intrinsic) factors^5^ will determine the selection of effective immune agents as a single compound or combination regimens. This becomes possible through the application of digital approaches, big data analysis and mathematical models, which go far beyond conventional techniques to answer important questions also in precision oncology.^12^

## 2 ARTIFICIAL INTELLIGENCE

With the advent of modern computing, many efforts are underway to replace, assist and augment human cognitive and analytic effort. It might not always be desirable, but it certainly allows addressing current objectives like the detection and readiness of complex immune biomarker. Such efforts are using AI attempting to create machine models for almost all aspects of human intelligence.^13^

Some common definitions name AI as the heading for machine learning (ML), of which among others like deep learning (DL) and convolutional neural networks (CNN) are usually considered further sub‐disciplines. However, different and sometimes conflicting definitions exist, of which none are wrong or correct. Both utilize machine cognition technologies with different levels of supervision and guidance by human experts having domain knowledge.^14^, ^15^ The development of AI relies to some degree on already existing and conventional expert knowledge creating rule sets or algorithms that support clinical decision‐making. AI's ability to describe current problems or anticipating problems of the future is also depending on the domain experts supporting such development.^16^

### 2.1 Machine learning

AI will be pivotal for the future practice of pathology and oncology. As mentioned above experts like pathologists and oncologists need to be involved in the development of AI‐based decision support to ensure a professional digitization of the medical practice and the generation of clinically relevant algorithms through their already existing knowledge and clinical experience. ML usually applies stochastic methods to analyze data sets creating independent and sometimes novel rules. ML is considered an attempt to support human experience and expert knowledge.^17^

A less ambitious goal is termed “narrow AI” which focuses on modeling presumably simpler tasks to support medical decision‐making. If successful, it will allow the transition from narrow AI to broader AI. This comprises also different layers of advanced algebra and topology,^18^ which describes the spatial relationship of immune cells and tumor cells and allows the functional cartography of tissue.^19^, ^20^, ^21^

Mathematical and computer science techniques allow domain experts but also others to extract relevant data from large data sets.^22^ Such algorithms can be trained or supervised by human experts or in this case by expert pathologists. ML can also assist experts in executing difficult and tedious tasks. An automated ML method will be able to consistently read multicolor immunohistochemistry or in‐situ hybridization images always in the same reliable manner,^23^, ^24^ producing the identical result over and over again. Such a machine‐assisted solution provides the basis for global comparability of even complex and larger data sets without otherwise non‐acceptable inter‐ and intra‐observer variability.^25^, ^26^

The field of AI‐based solutions and algorithms in pathology provide an increasing number of diagnostic and therapeutic decision support tools.^27^ The scanning and imaging of a whole (glass) slide has become a pivotal and prerequisite technology in histopathology that transfers conventional (analog) information into a high‐quality digital image to apply existing algorithms or solutions for further and spatial analysis.^28^ Only the precise, robust and reproducible diagnosis from tissue images will lead to an acceptance by pathologists with enough trust in such a disruptive technology. Already today, it is impressive how a computer can “read” a digitized image and “deliver” an accurate and quantitative interpretation, which goes beyond plain human eyeballing on a microscopic without computer assistance. Nevertheless, for the time being it is still necessary to confirm and validate any AI‐assisted diagnosis through a highly skilled and well‐trained pathologist concerning accuracy and plausibility.^29^

### 2.2 Visualization and explanation of data

Another important topic and prerequisite for the sustainable development of computational solutions in pathology as well as oncology is the use of curated data. Incorrect or inconsistent data will lead to incorrect conclusions and provide misleading decision paths. The cleaning and cleansing of data have a fundamental impact on the quality of such results. Therefore, data management and analytics needs to become an integrated part of the standard quality management and quality control throughout the entire workflow using computer‐assisted decision in clinical practice.^30^

An intuitive visual representation of complex data to pathologists and oncologists allows the understanding of the used algorithms and extracted information explaining the rationale behind AI‐based decision rules. The subject of topology as well as the cartography of the tissue microenvironment and its heterogeneity makes it now possible to further understand and visualize complex AI‐based solutions of multidimensional data sets.^31^ With the growing field of immune and combination therapies in precision oncology, a multitude of biomarker hypothesis will be integrated into topological networks, which will intuitively describe spatial relationships and relevant communication networks,^32^ possibly leading to relevant treatment decisions.^33^

AI tries to go beyond the “hidden secrets” or the “black box” nature of ML, which includes techniques such as convolutional neural networks, and DL. Those techniques allow an even deeper understanding and / or visualization of the complexity also of multidimensional features and cellular networks in heterogeneous tissue specimens to provide hypothesis or explanations of the results for pathologists' consumption and use.^34^ Expert pathologists still verify the concordance between the AI‐based decision rules and an already existing or accepted expert ground‐truth.^35^ As such explainable AI (X‐AI) or counterfactual explanations have developed as new disciplines in computational science with focus on explaining otherwise complex ML models, which are sometimes perceived as irrational or non‐conclusive. X‐AI tries to rationalize decision rules in analogy to what is already known by pathology and oncology experts. Naturally, human expert knowledge, which has been accumulated during many years of training and medical education and the rules of ML can be an area of conflict. However and in the ideal world, both approaches converge in their clinical validity with automated rule sets contributing significantly to machine‐based decisions in pathology and proving the sustainable correctness of medical and diagnostic practice.

## 3 PRECISION PATHOLOGY

Digital and computational pathology have become essential elements in translational research and transforming tissue‐based biomarker strategies and has put pathology back into the center of drug development or repurposing.^36^ Such technological innovation in the tissue biomarker space generates novel and big data, which is a continuous challenge for data analysts and clinical teams working to bring new drugs to market. The deployment and the adoption of precision pathology requires the preservation and scrutiny on the integrity of data to deliver novel and effective medicines. A multidisciplinary approach with the intense engagement of experienced pathologists, computer scientists, data analysts and biopharma specialists enables the discovery and validation of relevant tissue biomarker data to reach all desired biomarker endpoints from early phase clinical trials to market approval and across diverse therapeutic areas. The adoption of digital workflows will foster the best and future practice of pathology and the delivery of such relevant data and biomarker assays from preclinical discovery to clinical trials. Digital and computational pathology enables multidimensional image analysis that will become the standard for tissue biomarker delivery along with the continuous refinement of the laboratory workflow and implementation of machine intelligence‐supported technologies.^37^, ^38^, ^39^

### 3.1 Workflow automation

Precision Oncology or Personalized Cancer Medicine is based on the principle of optimized decisions proposing the most effective treatment for the certain cancer types or the individual subtype. This requires the development of complex assays that allow the identification of druggable targets in the tumor microenvironment as accurately and precisely as possible. The specificity and sensitivity of novel cancer biomarker tests are largely determined by the applied accuracy and precision of the respective analytical method. The necessary quality of the tissue sample(s) and the applied analytical method require the standardization of all steps from the pre‐analytical handling of the tissue specimen to the post‐analytical interpretation and scoring of the results. However, the potential variability associated with an individual patient sample must be monitored to ensure that only the correct test result is reported.

Preanalytical variables like different fixation times and processes still cause inconsistencies in the immune staining in many histopathology labs. Image analysis results cannot necessary be trusted entirely unless resolving such issues and implementing a vigorous quality management. Documented and proper sample handling, standardized processing of specimens including skilled embedding and sectioning, automated staining and scanning are important to develop and implement robust computational algorithms. These are the minimum requirements to maintain digital image consistency and robustness prior to any sustainable analysis and feature extraction. Anatomical or surgical pathologist together with the laboratory staff need perform such quality control measures throughout the entire workflow until this is also supported by computational solutions. Image analysis is an essential element of digital and computational pathology, and it demands special attention and competencies by the pathology staff and an understanding of the issue by the interacting computer and data scientists as well as software engineers.^40^ The number of publications and textbooks on these topics along with the advancement of necessary hardware technologies are steadily growing as well as the need for adequate image and data storage and processing capabilities.

The pivotal role of the pathologists is to master their responsibility from the bench to the bedside through their ability and growing experience and to implement and execute any type of (biomarker) assay robustly and sustainably, also in the routine diagnostic practice. As digital and computational pathology advances, the role of pathologists will transform and extend including the documented management of stringent quality control measures of the laboratory workflow and the handling of biomarker analytics that generates more and more data to stratify patients. Increasingly more insights that are therapeutic important will also come from multidimensional tests including spatial transcriptomics and other context‐driven information.^41^

### 3.2 Computational pathology

Recent advances in ML have accelerated computational pathology (CP) in medical research and clinical practice. Computational solutions will continue to support the diagnostic practice of pathology for yet well‐defined and selected tasks but in a reliable, consistent, and standardized way. Pathologists who are faced with an increased and complex workload will appreciate computational support.

The potential of ML techniques in pathology ranges from computer aided support for tasks that are simple but tedious like counting colored dots but also the discovery of innovative biomarker signature. Basic applications with simple dichotomous decisions are the detection of lymph node metastases or counting the density of mitotic or Ki‐67 positive tumor cells. CP is expected to increase the efficiency and precision in the entire tissue diagnostic workflow. First “simple” algorithms are already available and clinically viable. There are also computer‐modeling solutions that can extract sub‐visual morphological information relevant in personalized medicine and precision oncology.^42^ However, the increasing complexity of such applications requires large, curated, and cleaned datasets to leverage the full potential of CP in the future of pathology.^43^

Modern multiplexing technologies allow the simultaneous visualization of virtually hundreds of biomarker candidates on a single slide, visualizing the tumor heterogeneity.^44^, ^45^, ^46^ The standardized visualization of the spatially resolved complexity of immune and other markers requires a robust analysis of single and multiple (molecular or protein) marker molecules. This process starts with the digitization of images, followed by computer‐based image analysis, and further data breakdown through AI. As already stated before, CP leverages mathematical tools and implements data‐driven methods for large data sets and complex image interpretation in modern tissue diagnosis. The value proposition of CP as a part of digital pathology (DP) is especially high when clinical and pathology departments as well as informatics units work closely together on an interdisciplinary scale. CP will also become an integral part in the training of future pathologists, who will utilize their pathology and computational skills leading the field of CP and delivering an indispensable skill set for data‐related patient care.

### 3.3 Analysis of immune and tumor heterogeneity

As an integral part of CP image analysis allows the discovery and description of histomorphological features with diagnostic, prognostic and possibly predictive features relevant in the practice of precision medicine.^47^ The use of ML algorithms in CP along with advanced image analysis tools allows also the standardized assessment of known biomarker but likewise the discovery of novel immune signatures. Many relevant signatures in precision medicine are too subtle or not obvious to be recognized by human experts. The generation of a novel hypothesis from digital tissue images and supported by AI along with all available sets of big data will generate additional novel insights into the cancer biology and immune oncology.

With the clinical use of modern analytic and diagnostic tools such as multiplexed immune‐ and genotyping^48^, ^49^ along with AI comes a deeper understanding of the spatial relationship of immune and other cells in individual tissues, revealing the existing and relevant intra‐tumor heterogeneity which might have significant consequences for immune‐related and combination treatment options.^30^, ^50^ There are more and more warheads in the immune arsenal but there must be a scientific, financial, and medical rational for their clinical use.^51^, ^52^, ^53^

Many authorities, policy makers and payers demand the use of modern therapeutic modalities to be rationalized through a biomarker‐based and AI‐supported analytical and diagnostic strategy. The understanding of the tumor (immune) heterogeneity is a task of pathologists who advice the oncologist to select the best treatment option for each individual cancer patient. The microscopic inspection of the tumor, its associated microenvironment and surrounding normal tissue is no longer sufficient without the use of learning software solutions. Especially for advanced therapeutics (cell‐ and gene therapy) it is the only path towards a statement on the prognosis and possible predictions for the most effective treatment.^54^

A significant number of relevant biomarkers including proteins and genetic alterations have already been identified which guide therapeutic strategies and decisions in many tumor entities.^55^ Galon et al.^56^ demonstrated that the combination of two spatially resolved immune cell markers in different cancer tissue compartments show a better predictive value than each single marker alone.^57^ Such a development was only possible with a sound understanding of the cancer immunology, local tumor heterogeneity and an open mind towards computer‐assisted image analysis. Galon's group proposed a classification of a prognostic signature – the ImmunoScore ‐ based on the quantity and quality of immune infiltrates.^58^

Tumor and immune heterogeneity heavily influence the biology of each tumor and its response to treatment, including therapy resistance and some uncertainty of the histomorphological diagnoses. Genetic and epigenetic aberrations also influence the immune microenvironment and its plasticity and frequently vary from tumor entity to entity with or without previous therapy. Any failure of its identification may imply therapy relevant misinterpretations.^59^, ^60^, ^61^

### 3.4 Digital biomarker

Digital biomarker are generally defined as a combined software‐hardware solution to quantify measurable parameters that provide indications of a therapeutic response in a clinical environment. Digital biomarker also utilize data from different sources and measures to advance the understanding of a certain disease and guide the decision‐making also in the diagnosis and treatment of cancer.^62^ The idea of clinical immunotherapy is to (re)activate the immune system against uncontrolled tumor growth and spreading.^63^ This is an especially difficult task in certain cancer types with all the existing and known immune escape mechanisms^64^, ^65^, ^66^ that otherwise do not adequately respond to current strategies.^67^, ^68^, ^69^ Some tumor entities are anyway hard to treat for various and sometimes obvious reasons.^70^, ^71^, ^72^ Currently, there are no accepted biomarker signatures available for many immunotherapies.^73^ One of the known diagnostic challenge is to understand, visualize and determine the biologically relevant spatial relationship and communication network in the tumor microenvironment and retrieve actionable and clinically relevant information. Likewise, the analysis of multiple variables requires advanced technical tools and laboratory skills like high‐resolution image acquisition and analysis and the application of ML‐based algorithms to select patients for their best possible treatment option. Mathematical tools and AI‐based solutions will help to gain confidence in technically assisted decision making along with necessary clinical trials and experience. This is exemplified in the description of tumor infiltrating lymphocytes^74^ or the assessment of metastases in various tumors under immune therapy.^75^

## 4 PRECISION ONCOLOGY

The importance for advanced diagnostics to guide patient treatment decisions is growing fast. Table 1 describes the basic principles of AI and the use of ML in precision pathology as the foundation of precision oncology and their deliverables for best patient care.^76^

**TABLE 1:** Describes the basic principles of AI and precision pathology as the foundation of precision oncology. Their deliverables and effects will lead to a deeper understanding of the tumor biology and explaining even complex cancer networks that will better guide therapy selection for individual patients

	*Basic principles of action*	*Deliverables and effects*
**Artificial intelligence**		
Providing machine and deep learning solutions to pathology and tissue diagnostics	Developing learning algorithms and describing the contextual information of immune and tumor cells	Explaining relevant spatial and communication networks through tissue cartography
**Precision pathology**		
Using artificial intelligence to build and deploy predictive computational pathology	Establishing a consistent digital pathology workflow with standardized reporting	Automated image and data analyses delivering treatment relevant “Digital Biomarker”
**Precision oncology**		
Deep understanding of tumor biology and treatment relevant spatial immunity	Visualization of the tumor and immune heterogeneity to predict therapy response	Immune therapy selection through AI‐supported diagnostic decision tools

Patients that lack access to advanced cancer pathology guided by computer‐assisted diagnostic tools and expert decision boards are usually inadequately managed in their care. Expert pathologists are an increasingly scarce healthcare resource and therefore the “optimization” of their use especially important in times when their work is becoming more and more complex through AI‐based tools.^77^ Pathologists should engage with the AI development in pathology and its clinical implementation especially in precision oncology to assess its true value to the healthcare team.

Computer‐assisted decisions are also based on data from the real world or selected cohorts or named register like TCGA and are further supported by modalities like “systems medicine” and “*in‐silico”* modeling and simulation” approaches. AI will refine existing hypothesis of pathologists and immunologists to support diagnosis and therapy decisions.

### 4.1 Cancer immunotherapy

Cancer Immunotherapy has been named “Breakthrough of the Year” in 2013 and since then Nobel Prizes were awarded to scientists in this field. The clinical pertinence on the use of immune modulating agents like checkpoint inhibitors has been demonstrated in many clinical trials alone as a combination with other potent immune oncology (IO) drugs but also other non‐IO anti‐cancer agents.^78^, ^79^, ^80^ With the advancement and integrative use of analytical methods like immunohistochemistry (IHC), molecular pathology, and computational pathology it becomes increasingly possible to understand the morphological and immunological heterogeneity of individual tumors^54^ and better select appropriate clinical immunotherapies.^81^ Machine‐assisted diagnostic tools such as automated image analysis are available for the un‐biased and standardized assessment of multiple markers and simultaneously quantify the total numbers of different immune cells and in parallel the spatial relationships in different tumor compartments even on a single slide or image.^82^ Immune cells are a key component of predictive biomarker in the tumor immune microenvironment.^83^, ^84^

While technical solutions become available through the implementation of machine intelligence and digital biomarker, other existing barriers like some initially hesitant pathologists are fading away and digital pathology starts to spread. This is shown by the adoption of automated imaging solutions for primary diagnosis through available computational and imaging system. More and more pathology labs are starting to use digital pathology in their practice supported by guidelines, workshops, validation and accreditation procedures allowing the implementation of clinical grade digital pathology. Eventually it is computer science to assist diagnostic decision‐making and the acceptance of digital pathology especially in such a growing and demanding field such as precision oncology. By any means, it will increasingly be an interdisciplinary approach of domain experts from different biomedical fields and computer sciences with their focus on the well‐being and cure of cancer patients.

### 4.2 AI‐supported immunotherapy

Many genetic factors explicitly modulate the immune microenvironment and can influence the selection of IO drugs or the combination of IO molecules with non‐IO treatment regiments.^85^, ^86^, ^87^, ^88^, ^89^, ^90^, ^91^ Similarly, cancer‐associated fibroblasts (CAF) can also have a role in tumor progression and tissue remodeling secreting a wide range of humoral factors.^92^ Moreover, CAFs can be the reason for developing a resistance to guideline‐based therapies, as shown by Hirata et al. for BRAF‐inhibitor therapy in melanoma.^93^ The understanding of the tumor‐wide heterogeneity and the contextual information concealed with the individual tumor is important for treatment selection especially in the field of immune therapies.

The immunohistochemical evaluation of PD‐L1 is currently the diagnostic backbone for the response prediction of most IO therapies including checkpoint inhibitors, while routine histopathology still heavily relies on H&E stained slides and images. However, PD‐L1 testing is more complex than the familiar Ki‐67 or the current Her2/neu scoring due to different antibodies, different testing algorithms, and almost constantly changing cut‐offs for an increasing number of indications. The standard reporting of PD‐L1 scores requires skilled and trained pathologists always taking into account the substantial intra‐tumor heterogeneity.^94^, ^95^, ^96^, ^97^ There is already published evidence on the relevant heterogeneity of PD‐L1 expression between the primary tumor versus metastases^98^, ^99^ proving further evidence of the environmental (extrinsic) immune environment. More robust and validated digital biomarkers are needed that reflect the individual tumor immune microenvironment and its intrinsic (genomic) factors.^100^, ^101^, ^102^, ^105^ Further studies emphasized the relevance of cellular components in the immune system besides (epi)genetic biomarker. Both and in particular their spatial co‐existence have a significant prognostic value and need be included in therapeutic considerations.^103^, ^104^, ^105^, ^106^ Table 2 lists examples of AI‐assisted and digital biomarker in precision pathology and oncology with a special emphasis on immunotherapy.

**TABLE 2:** Lists examples of AI‐assisted decision support in precision pathology and those tools that have an even broader clinical impact in precision (immune) oncology. Digitale biomarker are also AI based but combine more digital and quantifiable characteristics from different sources that have a relevant impact on the clinical practice, here especially in the selection of immune therapies

	Precision pathology	Precision oncology
*AI‐assisted support*	Metastases detection^1^, ^35^ Multiplex immunohistochemistry^23^, ^24^, ^49^, ^51^ Whole sllide imaging^5^ Image analysis^27^, ^43^ PD‐L1 quantification^72^, ^94^, ^95^, ^99^	Large date extraction^22^ Multidimensional data analysis^31^, ^48^, ^49^ Mutation detection and analysis^6^, ^89^, ^107^ Spatial transcriptomics^41^ Drug development^36^
*Digitale Biomarker*	Disease diagnosis^2^ (Tumor) Immune infiltrates^58^, ^73^ Prognostic biomarker^30^, ^105^, ^106^	Personalized medicine and response prediction^23^, ^42^, ^47^ Cell‐ and Gene therapy^54^, ^68^ IO/non‐OI combination therapies^55^, ^64^, ^70^, ^77^ Immune escape prediction^63^, ^64^ Metastases assessment^39^, ^74^, ^98^

The future of precision cancer care will not only include the use of AI‐based algorithms in precision pathology and the diligent use of digital biomarker, but also major efforts to detect cancer earlier and with greater accuracy. The availability of larger data sets and a wider range of information from many sources (e.g. liquid biopsies, other imaging techniques) will help to identify the most effective treatments in a particular cancer and individual patient. Precision oncology will stratify many patients towards the most optimal cancer care right from the beginning which might be more effective, less costly and more likely to result in better overall outcomes.

## 5 CONCLUSION

**FIGURE 1:**
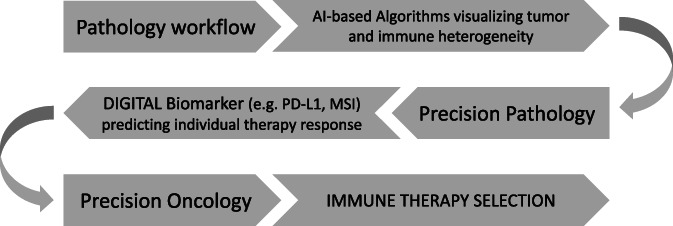
Simplified scheme of the proposed path from a regular diagnostic pathology workflow to delivering a precision oncology approach through the AI‐supported application of digital biomarker that allow the best possible response prediction of immune therapies.

Precision pathology will be the foundation and driver for precision oncology and immune therapies extending treatment regiments including oligo‐metastatic diseases or targeting the tumor microenvironment independent of the origin of the primary cancer. The basic principle is briefly summarized in Figure 1. Besides managing the “tumor data business”, also the technical and laboratory pathology workflow will change drastically leaving glass slides, conventional stains and eventually the light microscope behind and embracing 3D‐imaging including augmented and multiplex visualization techniques supported by AI. The implementation and execution of precision oncology including immune and combination therapies will be part of the medicine of the 21st century and pathologists will (co)lead such efforts embracing precision pathology.

## AUTHOR CONTRIBUTIONS

**Ralf Huss:** Conceptualization (lead); writing – original draft (lead); writing – review and editing (lead). **Johannes Raffler:** Conceptualization (supporting); validation (lead); writing – original draft (supporting); writing – review and editing (supporting). **Bruno Märkl:** Conceptualization (supporting); supervision (lead); validation (supporting); writing – original draft (supporting); writing – review and editing (supporting).

## CONFLICT OF INTEREST STATEMENT

The authors have stated explicitly that there are no conflicts of interest in connection with this article.

## ETHICS STATEMENT

This review does not include any human or animal studies or studies that require the approval of an institutional review board.

## Data Availability

Data sharing is not applicable to this article as no new data were created or analyzed in this study.
